# Proteomic Analysis of Crimped and Straight Wool in Chinese Tan Sheep

**DOI:** 10.3390/ani14192858

**Published:** 2024-10-04

**Authors:** An Shi, Sijia Ma, Zhuo Yang, Wei Ding, Jinyang Tian, Xin Chen, Jinzhong Tao

**Affiliations:** 1 College of Animal Science and Technology, Ningxia University, Yinchuan 750021, China; shian_1988@outlook.com (A.S.); masijia626@163.com (S.M.); jennyyangzhuo@126.com (Z.Y.); 2 Institute of Animal Science, Ningxia Academy of Agriculture and Forestry Sciences, Yinchuan 750002, China; dingwei398@163.com; 3 Ningxia Yanchi Tan Sheep Breeding Center, Ningxia Department of Agriculture and Rural Affairs, Wuzhong 751506, China; tyc20182023@163.com (J.T.); 17795037791@163.com (X.C.)

**Keywords:** proteomic, keratin, keratin-associated protein, proteomics, crimped wool, tan sheep

## Abstract

**Simple Summary:**

Tan sheep, a popular breed in China, exhibit distinctive crimped wool patterns that diminish with age, leading to a reduction in wool’s commercial value. This study examined the protein expression profiles of crimped and straight wool. We identified several key proteins, including KAP24-1, K84, K32, and K82, which are more abundant in crimped wool, and K6A, K27, K80, KAP16-1, KAP27-1, and trichohyalin (TCHH), which are more prevalent in straight wool. The findings provide insights into how these proteins influence wool morphology, potentially guiding improvements in wool quality for the textile industry.

**Abstract:**

Crimped wool in Tan sheep gradually transitions to straight wool after 35 days (the er-mao stage), which reduces its commercial value. To investigate the changes in wool proteins during this stage, we performed comparative proteomic analysis of the straight and crimped wool using tandem mass tag (TMT)-based quantification. The mean fur curvature (MFC) of crimped wool was significantly greater than that of straight wool (*p* < 0.001). We identified 1218 proteins between the two types of wool, including 50 keratins (Ks) and 10 keratin-associated proteins (KAPs). There were 213 differentially expressed proteins, including 13 Ks and 4 KAPs. Crimped wool showed relatively high abundances of KAP24-1, K84, K32, K82, and intermediate filament rod domain-containing protein (IRDC), whereas straight wool had relatively high abundances of K6A, K27, K80, KAP16-1, KAP27-1, and trichohyalin (TCHH). The expression levels of KAP16-1, KAP24-1, and KAP27-1 were related to the ratio of paracortex, which may be associated with wool crimp formation. Additionally, high expressions of TCHH, K27, and K6A in the inner root sheath (IRS) were linked to fiber fineness in straight wool. These findings provide insight into the overall expression and distribution patterns of Ks and KAPs, offering opportunities to improve wool quality and enhance its economic potential in the textile industry.

## 1. Introduction

**Figure 1:**
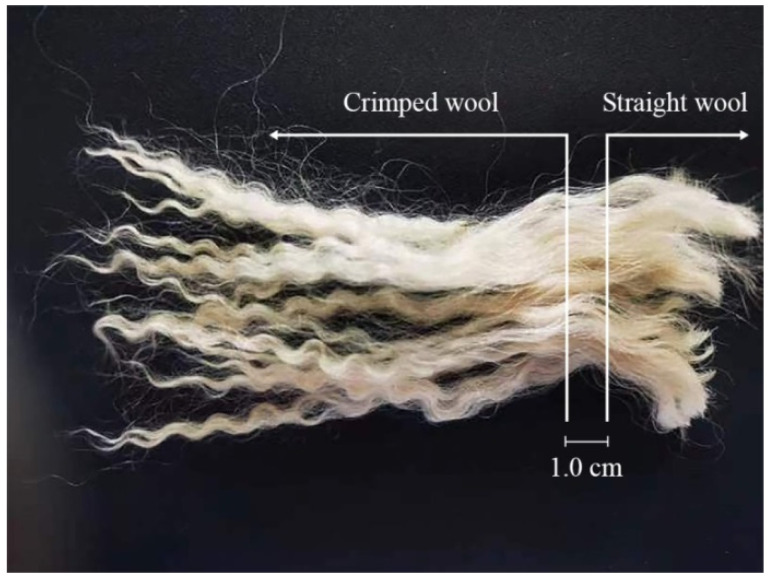
The Chuanzihua type of wool samples exhibit both straight and crimped characteristics.

Tan sheep are a popular breed for producing fur in Ningxia, China. Around 35 days of age, during the er-mao stage, the unique spike-type fur develops, known for its high quality [1]. At this time, the wool fibers are fine, soft, and elastic, with fiber lengths over 7 cm and about seven bends, forming a wave pattern known as “Chuanzihua” (Figure 1). The texture of er-mao fur is also at its best when sourced from Tan sheep skin, often used as a premium material for clothing. This wool holds substantial market value when commercialized [2]. However, during growth, the wool characteristics change, decreasing the value of the wool [3]. Understanding the factors that influence wool curvature in lambs may help maintain its quality and aesthetics as the sheep age. This can enhance the quality of wool products and economic value. The primary components of wool are keratin intermediate filament (KIF) and keratin-associated protein (KAP), which form over 90% of its composition [4]. KIFs create the wool’s framework, while KAPs, fill the spaces around them through disulfide bonds, forming the fiber matrix [5]. Consequently, the physical and mechanical properties of wool are largely determined by the content and distribution of these two components, significantly influencing the economic value of wool [6]. The mechanisms by which Ks and KAPs influence wool morphology are sought [7]. This strict spatiotemporal regulation of expression produces specific keratin content and distribution, ultimately leading to wool crimp [8]. Asymmetrical expression of *KRT27*, *KRT31*, *KRT35*, *KRT85*, and the trichohyalin gene (*TCHH*) in secondary follicles were associated with bulb deflection and follicle curvature [9]. The K38, KAP6, KAP7, and KAP8 families are lowly expressed in the orthocortex, while KAP16 and KAP19 are highly expressed in the orthocortex. The KAP4 and KAP9 families are highly expressed in the paracortex, collectively regulating wool curvature [10].

Recent advancements in proteomic analysis, particularly those using tandem mass tag (TMT)-based proteomics, have provided valuable insights into the protein expression profiles that influence wool morphology [11]. After comparing the proteomics of wool with different fiber diameters, Zhang discovered that the proportion of K85, KAP15-1, and KAP3-1 might be the key factors contributing to the differences in fiber diameter and could serve as molecular markers for distinguishing wool fineness [12]. Furthermore, in characterizing the skin protein profile at different fetal stages, proteomics identified 123 differentially abundant proteins (DAPs), which are closely related to metabolic and skin development pathways, as well as glycolysis/gluconeogenesis pathways. This indicates that fine-wool sheep regulate wool production through multiple pathways [13]. Additionally, proteomic analysis of three breeds with different wool phenotypes revealed significantly differentially expressed proteins, K75 and K38, which were suggested as potential markers for average curvature [14]. This approach allows for a comprehensive comparison of protein abundances between crimped and straight wool fibers, identifying key proteins involved in structural differences. Proteomics can provide a comprehensive view of protein expression patterns, which may be related to mechanisms such as spatiotemporal regulation and asymmetrical expression, and provide a molecular basis for understanding wool fiber morphology. The same breed of sheep, such as the Chuanzihua-type Tan sheep at the er-mao stage, can exhibit varying degrees of wool crimp, from crimped at the top and straight at the bottom, complicating the understanding of wool curvature mechanisms. Therefore, our hypothesis is that specific protein expression patterns are associated with wool crimp, influencing the structural and mechanical properties of the fibers. This study aims to identify and characterize these proteins and to elucidate their roles in wool morphology. These findings will provide valuable insights into how protein expression influences wool morphology, offering opportunities to improve wool quality and enhance its economic potential in the textile industry.

## 2. Materials and Methods

### 2.1. Wool Sample Collection

Wool samples were collected from four Chuanzihua-type Tan sheep at a breeding center in Yanchi, Ningxia, China. These four experimental animals were all 35-day-old healthy ram lambs with similar body conditions and weights. They were sired by the same ram, and all dams had the same parity. The management criteria included standardized feeding practices from birth to weaning, ensuring uniform nutrition and care for all lambs. Samples were taken from the posterior edge of the scapula on the left side of the sheep. The straight and crimped parts of the wool were separated based on their morphological characteristics (Figure 1) and the samples were stored at −80 °C.

### 2.2. Method of Trait Determination

The wool characteristics were measured, including the mean fiber diameter (MFD; μm), mean fiber curvature (MFC; /mm), fiber diameter standard deviation (FDSD; μm), and coefficient of variation of fiber diameter (CVFD; %). These parameters were determined following International Wool Textile Organization standardized methods formulated by the New Zealand Wool Testing Authority Ltd. (Napier, New Zealand). [15].

### 2.3. Wool Protein Extraction

Wool samples were ground repeatedly in a mortar containing liquid nitrogen to form a powder. A four-fold volume of lysis buffer (1% sodium dodecyl sulfate, 1% protease inhibitor, 15 mM dithiothreitol) was added to each sample, followed by ultrasonic lysis. The samples were centrifuged at 4 °C and 12,000× *g* for 10 min to remove the remaining debris. The supernatant was extracted, and the protein concentration was determined using a Bicinchoninic Acid Protein Assay Kit (Biyuntian BioTechnology, Shanghai, China).

### 2.4. Trypsin Digestion

Dithiothreitol was added to the protein sample, and the solution concentration was adjusted to 5 mM and reduced at 56 °C for 30 min. To prepare a final solution concentration of 11 mM, iodoacetamide was added to the sample and incubated at room temperature at 25 °C and in the dark for 15 min. Tetraethylammonium bromide (100 mM) was added to dilute the sample until the urea concentration was less than 2 M. The sample was digested twice with trypsin to protein mass ratios of 1:50 and 1:500; the former was subjected to protein digestion overnight, whereas the latter was digested for 4 h.

### 2.5. TMT Labeling

The tryptic peptides were desalinated using a Strata X C18 SPE column (Phenomenex, Torrance, CA, USA), followed by vacuum-drying. The peptides were dissolved in 0.5 M tetraethylammonium bromide, and then each channel and tandem mass tag reagent were label-matched, followed by incubation for 2 h at room temperature at 25 °C according to the instructions of the Tandem Mass Tag (TMT) Labelling Kit (Thermo Fisher Scientific, Waltham, MA, USA). Labeled peptides were desalinated and vacuum-dried in the same manner.

### 2.6. HPLC Fractionation

The peptides were fractionated using high-pH reversed-phase high-performance liquid chromatography (HPLC) on an Agilent 300Extend C18 (5 μm particles, 4.6 mm internal diameter, 250 mm length; Agilent Technologies, Santa Clara, CA, USA). The peptide fractionation gradient was 8–32% acetonitrile (pH 9.0) over 60 min to separate the 60 components. The peptides were combined into six components, followed by vacuum freeze-drying.

### 2.7. LC-MS/MS Analysis

Solvent A was composed of 0.1% formic acid and 2% acetonitrile, and solvent B was composed of 0.1% formic acid and 90% acetonitrile. Peptides dissolved in solvent A were separated using an EASY-nLC^TM^ 1200 liquid phase system (Thermo Fisher Scientific). The gradient settings were as follows: 0–2 min, 7–11% B; 2–52 min, 11–32% B; 52–56 min, 32–80% B; 56–60 min, 80% B. The flow rate was maintained at 500 nL/min. The separated peptides that were ionized after injection into the electrospray ionization were analyzed using Orbitrap Exploris^TM^ 480 Mass Spectrometry (Thermo Fisher Scientific) at an ion source voltage of 2.3 kV and a high-field asymmetric waveform compensation voltage of −45 V. The scan range and resolution were 400–1200 m/z and 60,000, respectively. The fixed starting point and scanning resolution of the secondary mass spectra were 110 m/z and 15,000, respectively. Data-dependent acquisition was performed, automatic gain control was set to 100%, the signal threshold was set to 5000 ions/s, the maximum injection time was set to auto, and the dynamic exclusion time for MS/MS scanning was set to 30 s to avoid repeated scans of the parent ions.

### 2.8. Database Searches

The resulting MS/MS data were processed using the MaxQuant search engine (v2.4.1.15), which were matched to the Universal Protein Resource (UniProt) database (Reference Proteome ID: UP000002356; https://www.uniprot.org/proteomes/UP000002356, accessed on 5 October 2022) with the taxon set to Ovis aries (23,108 entries). Trypsin/P was specified as the cleavage enzyme, allowing for up to four missed cleavages. The minimum length of peptides was set to seven amino acid residues, the maximum number of peptide modifications was set to five, mass tolerances for precursor ions were set to 20 and 5 ppm for the first and main search, respectively, and mass tolerance for fragment ions was set to 0.02 Da.

### 2.9. Statistical Analysis

Using the independent samples *t*-test in SPSS (v20.0, SPSS Inc. Chicago, IL, USA) to analyze the significance of differences in wool traits, the results are expressed as “mean ± standard deviation”. The relative quantitative proteins were calculated and imported into SIMCA (v14.1, Sartorius, Uppsala, Sweden) for principal component analysis (PCA) and orthogonal projection to latent structures discriminant analysis (OPLS-DA). To meet the assumptions of the *t*-test, the relative quantitative values of the proteins were log_2_-transformed to ensure the data followed a normal distribution. The screening criteria for differentially expressed proteins were variable importance in projection (VIP) > 1 and *p* < 0.05. After removing outlier protein data with inconsistent replicate values, the final key proteins were identified.

## 3. Results

### 3.1. Morphological Characteristics of Wool Samples

Wool collected from Tan sheep during the er-mao stage showed crimping at the top and straightness at the bottom. The MFC of crimped wool was 173% higher than that of straight wool (*p* < 0.001). There is no significant difference in MFD, FDSD, and CVFD between straight wool and crimped wool (*p* > 0.05) (Table 1).

### 3.2. Multivariate Statistical Analysis

**Figure 2:**
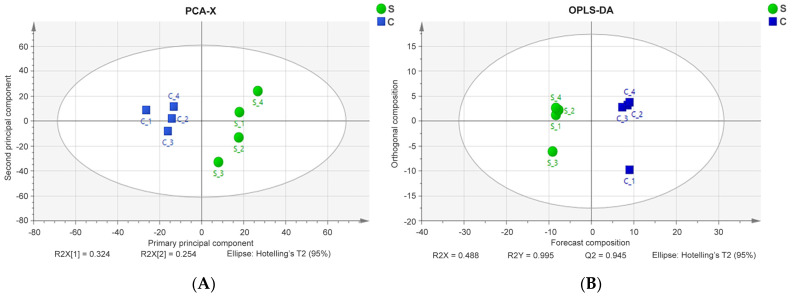
Score plots derived from PCA (**A**), and OPLS-DA analysis (**B**) based on LC-MS/MS data for crimped (blue squares) and straight (green circles) wool.

Multivariate statistical analysis was performed on eight samples from the crimped and straight wool groups using SIMCA v14.1 (Supplementary Table S6). All points were within the 95% confidence region of the PCA and OPLS-DA models. PCA revealed similarities and differences between the samples in the score plots (R^2^X = 0.578, Q^2^ = 0.138; Figure 2A). The crimped wool (blue squares) and straight wool (green circles) of the samples exhibited a concentrated distribution within groups and discrete patterns between groups. The OPLS-DA models also showed a trend towards high dispersion between the two groups (R^2^X = 0.488, R^2^Y = 0.995, Q^2^ = 0.945; Figure 2B). These results suggest that protein expression patterns differ significantly between crimped and straight wool.

### 3.3. Differential Expression of Proteins between Crimped and Straight Wool

In the wool samples from Tan sheep, we identified 1218 proteins including 50 Ks and 10 KAPs across the crimped and straight wool samples (*n* = 4 for each). (Supplementary Table S3). There were 213 differentially expressed proteins, including 13 Ks and 4 KAPs (Supplementary Table S4). After removing outlier protein data with inconsistent replicate values, the key proteins were finally identified. This study focused on the distribution and expression of Ks and KAPs, which led to the discovery that KAP24-1, K84, K32, K82, and IRDC were more abundant in crimped wool, while K6A, K27, K80, KAP16-1, KAP27-1, and TCHH were more abundant in straight wool. Two proteins were difficult to evaluate based on their peptide sequences because they showed substantial sequence homology (Table 2).

## 4. Discussion

Crimped wool naturally forms regular or irregular arcs along the length of fibers, with this curl typically measured as the MFC. During the er-mao stage in Tan sheep, the wool is crimped at the top and straight at the bottom. Actual measurement results show that the MFC of crimped wool is 173% higher than that of straight wool. The unique Chuanzihua type in Tan sheep provides a novel perspective for studying wool crimping. Quantitative proteomics based on TMT technology was used to evaluate the expression and distribution characteristics of Ks and KAPs, and their potential impact on wool curvature. A total of 13 key proteins were identified. Among them, KAP24-1, K84, K32, K82, and IRDC showed higher abundances in crimped wool, while K6A, K27, K80, KAP16-1, KAP27-1, and TCHH showed higher abundances in the straight wool. It was observed that both Ks and KAPs were highly expressed in each group.

The factors controlling wool fiber curvature remain elusive, and related research has revealed that the arrangement of the orthocortex and paracortex on either side of the fiber possibly leads to curvature [16]. Specifically, differential expression and distribution of various types of KAPs in the cortical layers play a role in altering wool structure [7]. In this study, we found that KAP16-1 and KAP27-1 were highly expressed in straight wool, while KAP24-1 was highly expressed in crimped wool. Although *KRTAP16-1* has not been previously investigated in sheep, variation in *KRTAP24-1* and *KRTAP27-1* has been studied in both sheep and goats [17,18,19], and variation in *KRTAP27-1* has been shown to influence wool traits, including wool growth in sheep [19] and fiber diameter in goats [20]. Since all three KAPs belong to high-sulfur proteins (HSPs) [18,21], and HSPs are preferentially expressed in paracortex cells [22,23], it is likely that straight wool contains a higher proportion of paracortex cells, while crimped wool contains fewer. Therefore, we hypothesize that varying ratios of paracortex may alter the arrangement of the orthocortex and paracortex, thereby influencing wool curvature. Additionally, K32, K82, and K84 were highly expressed in crimped wool. In crimped human hair, K82’s asymmetric expression in the precortex has been proposed as a possible mechanism behind curl formation [24]. In addition to the differential distribution of Ks and KAPs in the cortex, the structural arrangement of KIFs may also influence wool curvature [25]. Chen found that *KRT32* exons in Gansu Alpine fine-wool sheep have six SNPs (g.21455859, g.21455953, g.21455976, g.21456106, g.21460798, and g.21460884) that are significantly associated with MFC, including three synonymous mutations and two missense mutations [26]. Meanwhile, SNPs in the first exon of *KRT84* were also found to be significantly associated with MFC [27]. Mutations in the *KRT32* and *KRT84* genes alter the protein structure, which may consequently change the structural arrangement of KIFs and thus affect MFC.

K27 [28], K6A [29], and TCHH [30], which were highly expressed in straight wool, were specifically expressed in the IRS. There is currently limited research on whether they are also expressed in wool fibers. K72, which is expressed in the IRS, has also been found to be expressed in wool fibers [28,31], suggesting that IRS-specific genes may also be expressed in wool fibers. Research has shown that the unique cross-bridging structure of TCHH can connect Ks, providing fiber strength [32]. It is hypothesized that TCHH, K27, and K6A are involved in establishing the initial differentiation of IRS structures, which ultimately leads to fiber straightening. In this study, although there were no statistically significant differences in MFD, FDSD, and CVFD between straight and crimped wool, there was a trend towards a smaller average fiber diameter and a more consistent diameter distribution in straight wool. This trend may still provide meaningful insight into the higher expression levels of TCHH, K27, and K6A in straight wool. There have also been relevant studies on the genetic aspects, showing that *KRT27* is significantly more highly expressed in the skin tissues of superfine-wool sheep compared to fine-wool sheep [33]. Studies have reported that the *TCHH* SNP is associated with wool fineness [34]. Research on non-coding RNAs has identified *TCHH* as a target gene in a ceRNA network that regulates wool fineness [35]. K80, localized in epithelial cells of wool [36], may be present in both the outer root sheath (ORS) or IRS during follicle differentiation, and further research is needed to determine whether its link with TCHH alters fiber structure.

Additionally, IRDC, a protein with an α-helical rod domain characteristic of intermediate filaments, remains unexplored regarding its role in wool function. This protein could potentially be involved in the structural integrity and mechanical properties of wool, but further research is necessary to confirm its function. Similarly, two other key proteins identified are currently uncharacterized. Their presence in both crimped and straight wool suggests they may play a role in wool morphology. It is speculated that these proteins might belong to the Ks or KAPs families; however, they have not yet been identified. Investigating these uncharacterized proteins could lead to new insights into wool fiber development and structure. The functional mechanisms of TCHH, in combination with specific Ks like K82 and K6A, will be explored further. This will help clarify their roles in fiber straightening and strength, especially given TCHH’s unique cross-bridging capabilities that link Ks and provide structural stability to the wool fiber. These investigations will not only expand our understanding of wool biology but also open up potential avenues for improving wool quality through targeted breeding or genetic interventions.

## 5. Conclusions

The expression levels of KAP16-1, KAP24-1, and KAP27-1 were related to the ratio of the paracortex, which may be associated with wool crimp formation. Additionally, high expressions of TCHH, K27, and K6A in the IRS were linked to fiber fineness in straight wool. These findings highlight the distinct protein expression profiles that underlie the structural differences between crimped and straight wool, offering valuable insights into the overall expression and distribution patterns of Ks and KAPs, with potential implications for improving wool quality in the Tan sheep.

## Figures and Tables

**Table 1:** Descriptive statistics of morphological characteristics of straight and crimped wool in the er-mao stage of Tan sheep.

Wool Traits	Straight Wool (*n* = 4)	Crimped Wool (*n* = 4)	*p*-Value
MFC, °/mm	27.65 ± 1.39	75.45 ± 3.02 ***^1^	<0.001
MFD, µm	27.43 ± 3.13	27.46 ± 1.30	0.984
FDSD, µm	7.63 ± 1.48	8.88 ± 1.21	0.239
CVFD, %	26.73 ± 2.81	27.75 ± 3.71	0.675

^1^ In the same row, *** *p* < 0.001, *p* > 0.05 represents no significant difference.

**Table 2:** The differential expression of Ks and KAPs in straight and crimped wool.

UniProt ID ^1^	Protein Name	Molecular Weight ^2^, kDa	Fold Change, S/C ^3^	*p*-Value	VIP
W5Q0U7	K27	50.10	1.396	0.010	1.478
P22793	TCHH	201.10	1.534	0.005	1.530
W5Q350	KAP16-1	55.20	1.356	0.005	1.569
W5NS40	KAP27-1	22.90	1.313	0.023	1.390
W5Q6B8	K6A	57.30	1.308	0.049	1.276
W5Q6V9	K80	48.10	1.309	0.004	1.596
W5Q2K6	Uncharacterized ^4^	20.90	1.639	0.003	1.673
J7GWF2	KAP24-1	27.90	0.771	0.018	1.457
W5Q6J4	K84	64.10	0.901	0.014	1.455
W5Q5A7	K32	45.00	0.843	0.049	1.277
W5P2K5	IRDC	43.90	0.815	0.003	1.594
W5Q6H2	K82	56.70	0.788	0.001	1.631
W5P4I1	Uncharacterized	19.10	0.854	0.016	1.555

^1^ UniProt ID represents the format name used to retrieve the corresponding protein in the Universal Protein Resource database. ^2^ The molecular weight of a protein is determined by the composition of its amino acids, each of which has a different molecular weight. ^3^ The term “S/C” represents the comparison of straight wool with crimped wool in proteomic analysis, used to measure the differential protein expression between these two experimental groups. When the fold change is >1, it indicates that the corresponding protein is highly expressed in straight wool, while when the fold change is <1, it indicates that the protein is highly expressed in crimped wool. ^4^ Uncharacterized means a type of protein entry in the UniProt database that has not been thoroughly studied yet. These proteins may be newly discovered or less researched, and their functions, structures, and biological activities are not yet fully understood.

## Data Availability

The dataset is available upon request from the authors.

## Supplementary Materials

The following supporting information can be downloaded at https://www.mdpi.com/article/10.3390/ani14192858/s1, Table S1: Analysis data of wool traits; Table S2: The LC-MS/MS analysis identified a total of 1218 proteins; Table S3: A total of 50 Ks and 10 KAPs were identified from the total proteins; Table S4: A total of 213 differentially expressed proteins were identified; Table S5: Further reference to annotated sequences confirmed 13 key proteins; Table S6: The complete data for PCA and OPLS-DA multivariate statistical analysis plots.
