# Supplementary material for: Proteomic Analysis of Crimped and Straight Wool in Chinese Tan Sheep
Source: Animals (Basel). 2024 Oct 4;14(19):2858. doi: 10.3390/ani14192858 (PMC11482551; doi:10.3390/ani14192858)
Supplement: Supplementary file 1 [file animals-14-02858-s001.zip › Supplementary materials list.docx]

# Supplementary Material

**Supplementary Table list**

**Table S1.** Analysis data of wool traits.

**Table S2.** The LC-MS/MS analysis identified a total of 1218 proteins.

**Table S3.** 50 keratins and 10 keratin-associated proteins were identified from the total proteins.

**Table S4.** A total of 213 differentially expressed proteins were identified.

**Table S5.** Further reference to annotated sequences confirmed 13 key proteins.

**Table S6.** The complete data for PCA and OPLS-DA multivariate statistical analysis plot.
